# Identifying prognostic signature in ovarian cancer using DirGenerank

**DOI:** 10.18632/oncotarget.18189

**Published:** 2017-05-25

**Authors:** Jian-Yong Wang, Ling-Ling Chen, Xiong-Hui Zhou

**Affiliations:** ^1^ College of Informatics, Huazhong Agricultural University, Wuhan 430070, P.R. China

**Keywords:** DirGenerank, ovarian cancer, prognosis, biomarker, drug target

## Abstract

Identifying the prognostic genes in cancer is essential not only for the treatment of cancer patients, but also for drug discovery. However, it's still a big challenge to select the prognostic genes that can distinguish the risk of cancer patients across various data sets because of tumor heterogeneity. In this situation, the selected genes whose expression levels are statistically related to prognostic risks may be passengers. In this paper, based on gene expression data and prognostic data of ovarian cancer patients, we used conditional mutual information to construct gene dependency network in which the nodes (genes) with more out-degrees have more chances to be the modulators of cancer prognosis. After that, we proposed DirGenerank (Generank in direct netowrk) algorithm, which concerns both the gene dependency network and genes’ correlations to prognostic risks, to identify the gene signature that can predict the prognostic risks of ovarian cancer patients. Using ovarian cancer data set from TCGA (The Cancer Genome Atlas) as training data set, 40 genes with the highest importance were selected as prognostic signature. Survival analysis of these patients divided by the prognostic signature in testing data set and four independent data sets showed the signature can distinguish the prognostic risks of cancer patients significantly. Enrichment analysis of the signature with curated cancer genes and the drugs selected by CMAP showed the genes in the signature may be drug targets for therapy. In summary, we have proposed a useful pipeline to identify prognostic genes of cancer patients.

## INTRODUCTION

Ovarian cancer, one of the most common malignant cancer [1], is urgently needed to improve outcomes through developing new therapy. Identifying the prognostic genes in cancer is essential not only for the treatment of cancer patients, but also for drug discovery. Thus it's of great interest to select prognostic genes in ovarian cancer [2]. Using gene expression data of ovarian cancer patients, many studies have succeeded in selecting gene signatures with prognostic relevance [3–5]. However, most of the signatures which are selected according to the genes’ statistical relevance with the prognostic risks of cancer patients perform poorly in independent data [6], which may be caused by the high heterogeneity of cancer [7]. In this situation, the selected genes whose expression levels are statistically related to prognostic risks may be passengers. Therefore, it is of great importance to identify the driver genes in cancer [8], for the purpose of selecting prognostic genes with therapeutic value.

Cancer is a complex disease which may result from the alterations of multigenes [9]. In addition, the genetic alterations do not occur separately, but depend on each other [10, 11]. Thus, if we could infer the gene dependency relations in the progression process of cancer patients, it would be of great help to identify the driver genes. There are also some works succeeding in identifying signature in ovarian cancer concerning the co-expression relations or other undirected relations among lincRNAs and genes [12–14]. However, these works can't reveal the gene regulatory relation behind the phenotypic change of cancer patients. In our previous work [15], based on gene expression data and clinical data of cancer patients, we proposed a new method to construct a gene dependency network using conditional mutual information. The network has been demonstrated to be able to uncover the biological mechanism in the process of phenotypic change.

In this work, we apply the above method to infer the gene dependency network in the prognosis process of ovarian cancer, using gene expression data and clinical data of ovarian cancer patients. In this network, an edge from Node *A* to Node *B* denotes the mutual information between expression levels of gene *A* and the prognostic risks of cancer patients is significantly dependent on the gene expression levels of gene *B*. Therefore, the nodes (genes) with more out-degrees have more chances to be the modulators of cancer prognosis and these nodes are more likely to be drivers. Pagerank [16], which is invented by GOOGLE, succeeded in ranking the important webpages on the Internet through the links among these web pages. Generank [17], which is based on Pagerank, has been proposed to prioritize genes in an undirected biological network. Here, an extended version of Generank (DirGenerank), which can be used in a direct biological network, is proposed. The DirGenerank algorithm concerns both the gene dependency network and genes’ correlations to prognostic risks to make sure the selected prognostic signature is more likely not only to be drivers, but also to be prognostic. In order to validate the clinical value of the prognostic genes, we first validated the distinguishing capability of the prognostic genes in five testing data sets. After that, the independence of the prognostic genes’ prognostic value from clinical variables was investigated by multivariate Cox regression. And we also tested the robustness of our method by investigating the stability of the prognostic genes with different parameters in the pipeline. Finally, we used enrichment analysis of these genes with curated cancer genes to validate our method. In addition, based on the prognostic genes, we selected drugs using CMAP (The connectivity map) [18, 19] to test whether the genes in the signature are good candidates for drug targets.

## RESULTS

### Pipeline to identify prognostic genes

**Figure 1:**
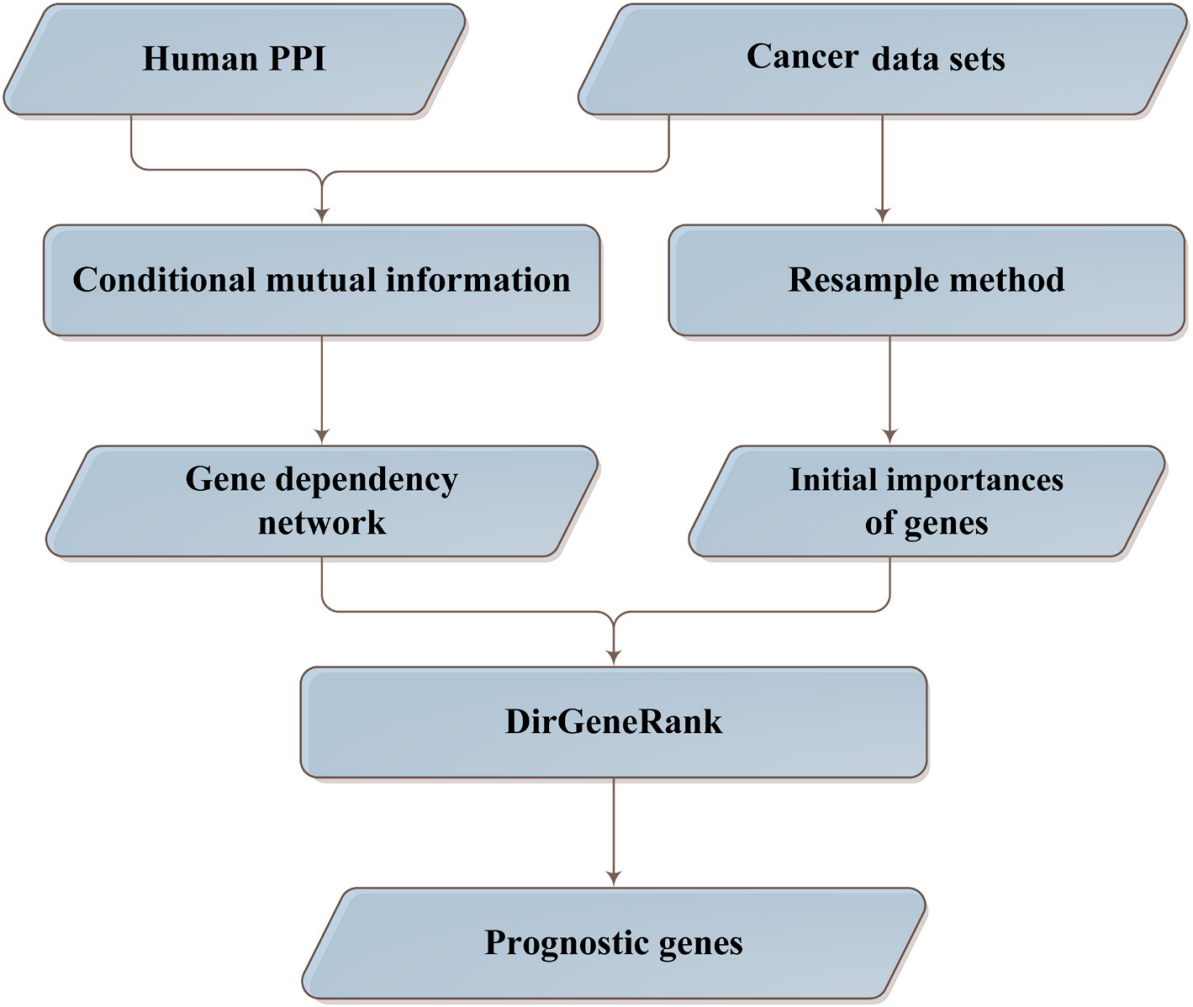
Pipeline to identify prognostic genes

The prognostic genes are usually obtained by calculating the statistical relation of gene expression levels and the prognostic risk of cancer patients. However, because of tumor heterogeneity, these prognostic genes are usually not robust in independent data sets. Based on the hypothesis that the biological network can facilitate the identification of driver genes in cancer prognosis, we proposed a new pipeline to identify prognostic genes, which is based on gene expression data and prognostic data of cancer patients. The pipeline is shown in Figure 1.

First of all, based on gene expression data and clinical information of cancer patients (ovarian cancer in this work), gene dependency network was constructed using conditional mutual information. In this network, a high out-degree means the mutual information of many genes with the clinical information (prognostic risk in this work) is dependent on it. Therefore, the higher the out-degree of the gene is, the more likely the gene is to be a driver.

After that, resample method (Method) was applied to calculate the prognostic capability of each gene, which would be used as the initial importance of the genes in the next stage.

Then, the DirGenerank algorithm (Method), which is a modified Pagerank algorithm, was proposed to identify the prognostic genes in ovarian cancer. The algorithm contains two inputs: a matrix describes the regulatory relation among genes (gene dependency relation in this work), and a vector describes the initial importance of the genes in the network. The output of the algorithm is the importance of the genes after n iterations. It is worth noting that the importance of the genes is dependent on the initial importance and the gene dependency network. The former is the statistical correlation between gene and phenotype (prognostic risk), and the latter is the gene dependency relations among all the genes. In addition, the two parts are weighted by a constant d. In this work, we set d as 0.7, which is the same as a previous work did [20].

By the above pipeline, the prognostic genes, which may be more likely to be driver genes, may be identified.

### The prognosis related gene dependency network

**Figure 2:**
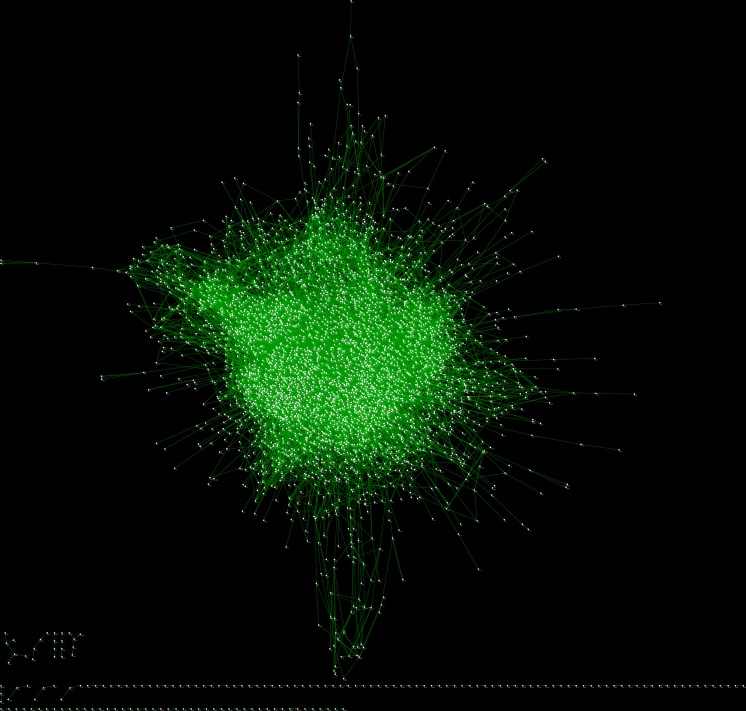
The dependency network in ovarian cancer

In our previous work [15], we demonstrated that gene dependency network based on breast cancer data set is able to uncover the gene dependency relation in cancer prognosis. Here, we applied it in ovarian cancer data set to construct the gene dependency network in ovarian cancer using TCGA (Methods), in which nodes are genes and a direct edge from node *A* to node *B* means the mutual information between gene *B* and prognostic risk of cancer patients is significantly dependent on gene *A*. Therefore, the nodes with high out-degrees indicate more possibilities to be modulators of cancer prognosis in ovarian cancer. The network was shown in Figure 2.

In this network, there are 3995 nodes and 19791 edges (Supplementary Table 1). The average neighbours of the nodes are 9.21. Furthermore, the power-law fit of the nodes’ degrees with the number of the nodes (with the according degrees) shows both the nodes’ in-degree and out-degree in the network fit power law distribution very well. The correlation and R-square of the nodes’ in-degree's fitting are 0.80 and 0.88 respectively (Supplementary Figure 1a), and the correlation and R-square of the nodes’ out-degree's fitting are 0.77 and 0.87 (Supplementary Figure 1b).

We also investigated some gene dependency relation in the network by literature survey. As we know, BLC2 (Entrez ID: 4609) and MYC (Entrez ID: 596) are both involved in the pathway of apoptosis and thus influence the development [21] and prognosis of cancer [22]. It is more interesting that MYC and BCL2 may act synergistically to promote the generation of cancer cells [23]. In the meanwhile, BCL2 is significantly dependent on MYC with a p-value of 0.013 and MYC is also dependent on BLC2 significantly, with a p-value of 0.032 in our network. ABAC1 (Entrez ID: 20) mediate the drug- resistance in ovarian cancer [24], and BRCA1 is the most famous susceptibility gene in ovarian cancer [25]. According to our gene dependency network, the mutual information of ABAC1 to prognostic risk of ovarian cancer patients is dependent on BRCA1 (The p-value of the edge from BRCA1 to ABCA1 is 0.045).

In conclusion, topology analysis of our gene dependency network and case study of the gene dependency network shows our network could be used to reveal the gene dependency relation in cancer prognosis of ovarian cancer.

### The prognostic genes selected in ovarian cancer

Using the pipeline described above, based on the gene dependency network and the prognostic capability of all the genes (Supplementary Table 2) evaluated by resample method, DirGenerank was used to identify the prognostic genes in ovarian cancer. According to the importance calculated by the algorithm, 1% of 3995 nodes in the network, that is, 40 genes were obtained as prognostic genes (Table 1).

**Table 1:** The prognostic genes identified by our pipeline

Gene Id	Cox coefficient	Stability	Gene symbol	Description
**3174**	−0.611	397	HNF4G	hepatocyte nuclear factor 4, gamma
**2250**	−0.542	400	FGF5	fibroblast growth factor 5
**4802**	−0.517	375	NFYC	nuclear transcription factor Y, gamma
**27329**	−0.465	400	ANGPTL3	angiopoietin-like 3
**22955**	−0.460	399	SCMH1	sex comb on midleg homolog 1 (Drosophila)
**3026**	−0.430	393	HABP2	hyaluronan binding protein 2
**1453**	−0.422	358	CSNK1D	casein kinase 1, delta
**3607**	−0.413	392	FOXK2	forkhead box K2
**3207**	−0.332	400	HOXA11	homeobox A11
**2113**	−0.281	240	ETS1	v-ets erythroblastosis virus E26 oncogene homolog 1 (avian)
**636**	−0.264	386	BICD1	bicaudal D homolog 1 (Drosophila)
**362**	−0.235	281	AQP5	aquaporin 5
**8706**	−0.204	314	B3GALNT1	beta-1, 3-N-acetylgalactosaminyltransferase 1 (globoside blood group)
**3918**	−0.203	399	LAMC2	laminin, gamma 2
**3570**	−0.193	299	IL6R	interleukin 6 receptor
**6361**	−0.185	346	CCL17	chemokine (C-C motif) ligand 17
**1381**	−0.132	399	CRABP1	cellular retinoic acid binding protein 1
**10753**	−0.130	381	CAPN9	calpain 9
**8856**	−0.0935	0	NR1I2	nuclear receptor subfamily 1, group I, member 2
**10563**	−0.0762	31	CXCL13	chemokine (C-X-C motif) ligand 13
**8503**	−0.0669	0	PIK3R3	phosphoinositide-3-kinase, regulatory subunit 3 (gamma)
**554**	0.0142	0	AVPR2	arginine vasopressin receptor 2
**8877**	0.0616	0	SPHK1	sphingosine kinase 1
**646**	0.10637585	391	BNC1	basonuclin 1
**1746**	0.161	97	DLX2	distal-less homeobox 2
**10894**	0.190	395	LYVE1	lymphatic vessel endothelial hyaluronan receptor 1
**2173**	0.193	319	FABP7	fatty acid binding protein 7, brain
**6622**	0.209	391	SNCA	synuclein, alpha (non A4 component of amyloid precursor)
**5733**	0.236	399	PTGER3	prostaglandin E receptor 3 (subtype EP3)
**27129**	0.253	398	HSPB7	heat shock 27kDa protein family, member 7 (cardiovascular)
**56914**	0.262	320	OTOR	otoraplin
**344**	0.296	399	APOC2	apolipoprotein C-II
**11027**	0.310	396	LILRA2	leukocyte immunoglobulin-like receptor, subfamily A (with TM domain), member 2
**3036**	0.350	378	HAS1	hyaluronan synthase 1
**8904**	0.367	396	CPNE1	copine I
**324**	0.406	385	APC	adenomatous polyposis coli
**7082**	0.456	400	TJP1	tight junction protein 1 (zona occludens 1)
**2022**	0.4778	356	ENG	endoglin
**1176**	0.510	397	AP3S1	adaptor-related protein complex 3, sigma 1 subunit
**3562**	0.887	329	IL3	interleukin 3 (colony-stimulating factor, multiple)

For each gene, cox coefficient is the average coefficient of 400 cox proportional hazards regressions in the 400 resampling data sets. The stability of a gene is set as the times when it's significant in 400 cox proportional hazards regressions.

From this table, we can see that the prognostic genes obtained by using our method not only contain the genes whose expression levels are significantly correlated with prognostic risks, but also contain genes whose expression levels are not directly related to the prognostic risks in statistics. The latter ones can be identified by our method because they may modulate many genes (with high out-degrees) to influence the prognosis of cancer patients, and thus they could be prioritized by DirGenerank. For example, PIK3R3 (Entrez ID: 8503) is correlated with the prognostic risks of cancer patients in none of the 400 resamples, but it is identified by our method. In addition, it has been reported that mutation of PIK3R3 is related to ovarian cancer [26] and PIK3R3 is a potential therapeutic target for ovarian cancer [27].

We also investigated the intersection between our prognostic genes and the curated cancer gene (Sanger and COSMIC). Hypergeometric test shows the intersection is significant, with a p-value of 0.0025. In the meanwhile, the most significant genes (40 genes) calculated by resample method were set as control signature (Supplementary Table 3) to validate our method. Overlap of the genes in control signature and the cancer genes was also investigated, but the p-value of hypergeometric test is 0.4111. This result indicates the prognostic genes identified by the new pipeline may be more likely to be real cancer genes, compared with the traditional method.

### Functional annotation of the prognostic genes

Enrichment analysis of the prognostic genes with the KEGG pathway was done by GSEA [28]. The KEGG pathways in which the prognostic genes were significantly involved were listed in Table 2.

**Table 2:** Function annotation of the prognostic genes

Pathways	p-value	FDR q-value
**Pathways in cancer**	9.63E-06	1.79E-03
**Cytokine-cytokine receptor interaction**	8.64E-05	8.03E-03
**Jak-STAT signaling pathway**	3.39E-04	2.10E-02
**Chemokine signaling pathway**	6.14E-04	2.86E-02
**Regulation of actin cytoskeleton**	8.90E-04	2.95E-02
**Endometrial cancer**	9.53E-04	2.95E-02
**Colorectal cancer**	1.35E-03	3.50E-02
**Renal cell carcinoma**	1.72E-03	3.50E-02
**Melanoma**	1.77E-03	3.50E-02
**VEGF signaling pathway**	2.02E-03	3.50E-02
**Fc epsilon RI signaling pathway**	2.18E-03	3.50E-02
**Small cell lung cancer**	2.46E-03	3.50E-02
**Apoptosis**	2.70E-03	3.50E-02
**Hematopoietic cell lineage**	2.70E-03	3.50E-02
**Gap junction**	2.82E-03	3.50E-02
**Fc gamma R-mediated phagocytosis**	3.26E-03	3.79E-02

From this table, it is clear that 16 pathways are significant (FRD < 0.05). It needs to mention that ‘Pathway in Cancer’ is the most significant one, with FDR of 1.79e-03. What is more interesting is that several sub-pathways in ‘Pathway in cancer’ were also significantly enriched, such as ‘Cytokine-cytokine receptor interaction’, ‘Jak-STAT signaling pathway’ and ‘Apoptosis’. In addition, 5 out of the other pathways are pathways in specific cancers (endometrial cancer, colorectal cancer, renal cell carcinoma, melanoma, small cell lung cancer). ‘Chemokine signaling pathway’ and ‘VEGF signaling pathway’ are also significant, and it was also reported that Chemokine signaling system may be an important therapy target for ovarian cancer [29]. In addition, VEGF signaling pathway has also been validated to be a therapeutic target for cancer treatment [30]. In a word, most of the pathways in which our prognostic genes are involved are either cancer-related pathways or targets for cancer therapy.

### Survival analysis of the ovarian cancer patients using the prognostic genes

As reported before, most of the prognostic genes derived from gene expression data are confronted with poor generalization [31]. In order to validate the prognosis capability of our prognostic genes, we constructed prognosis model and tested it in five ovarian cancer data sets, with a number of more than 2,000 samples.

**Figure 3:**
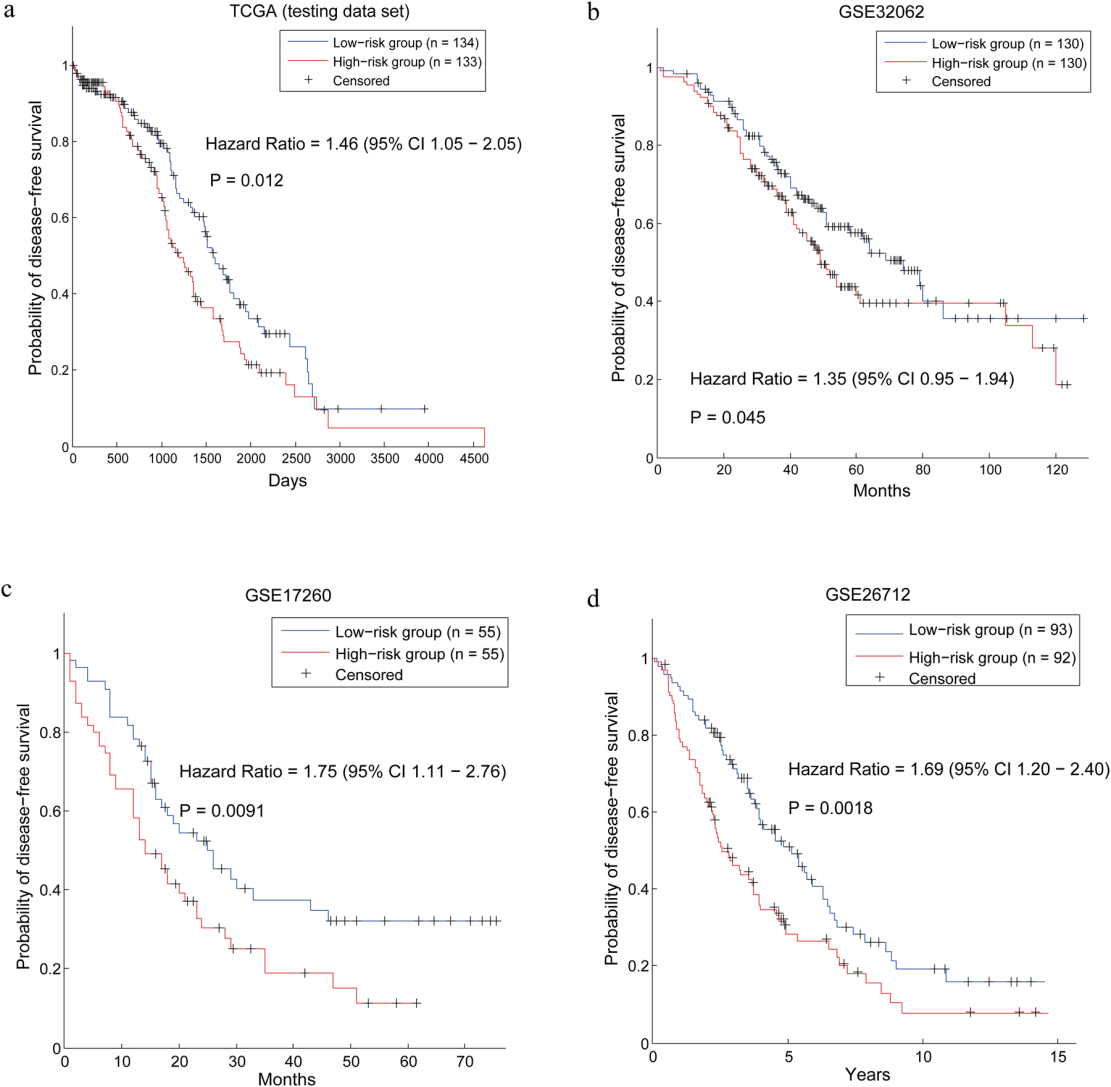
Survival analysis of the patients divided by the prognostic genes in four data sets

First of all, the death-risk score of each cancer patient was calculated using the 40 prognostic genes. After that, the patients in each data set were divided into two groups based on their risk scores (Method). Then survival analysis was used to test whether there are significant differences in the true death risks between the two groups of patients. In the training data set (300 patients in TCGA), survival analysis shows the hazard ratio (HR) of the two groups is 3.96 (95% confidence interval: 2.80 – 5.61) and the p-value of the log-rank test is 1.11e-16 (Supplementary Figure 2). In the testing data set (267 patients in TCGA), the HR of the two groups divided by our method is 1.46 and p-value is 0.012 (Figure 3a).

**Figure 4:**
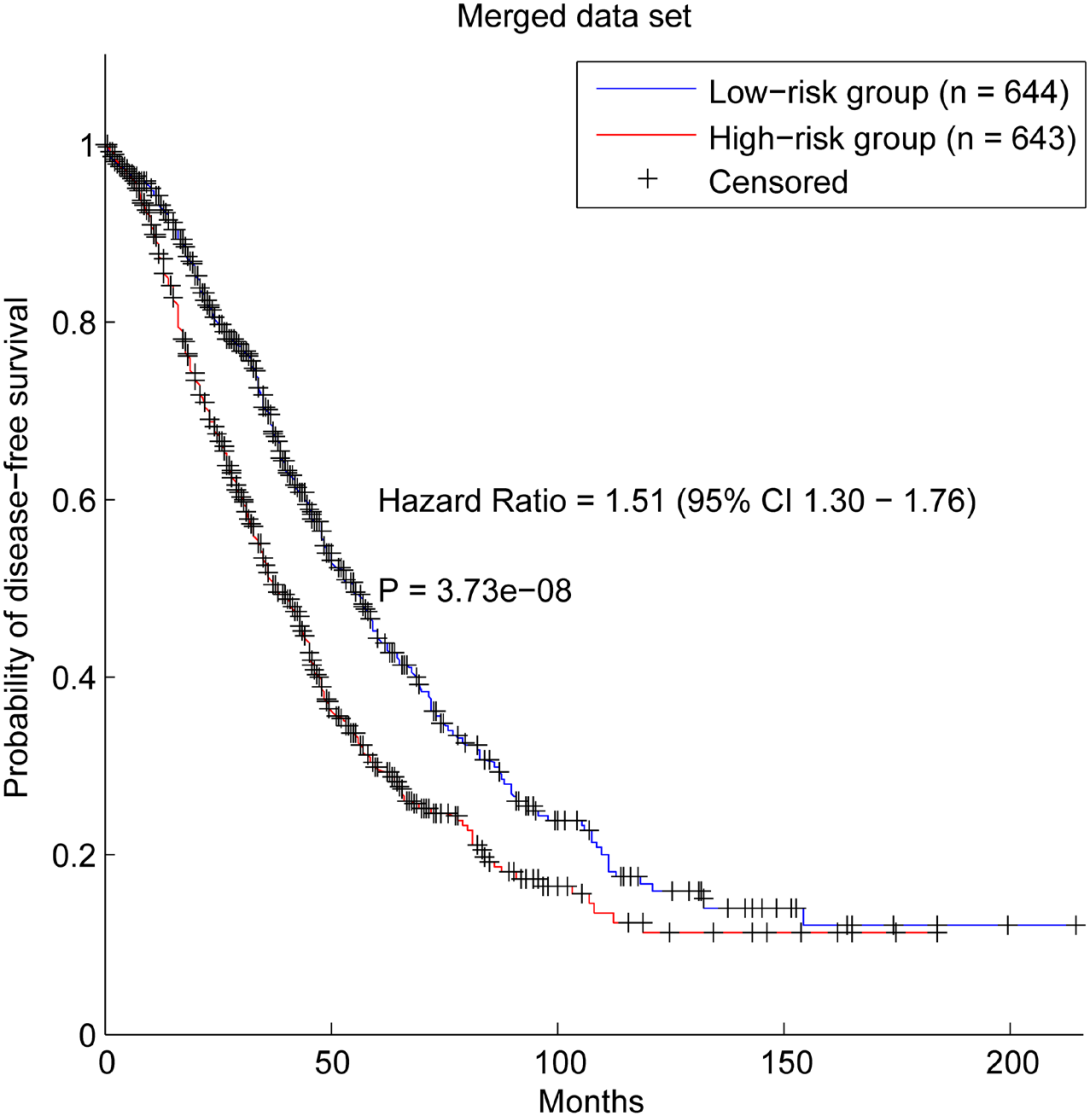
Survival analysis of patients divided by the prognostic genes in the merged data set

Furthermore, we validated our method in three independent data sets, which are GSE32062, GSE17260 and GSE26712. The results of our method in the three data sets are with HR (p-value) of 1.35 (0.045), 1.75 (0.0091) and 1.69 (0.0018) respectively (Figure 3b-3d). In a previous work [32], 1287 ovarian cancer samples were collected to validate survival-associated biomarkers. In this work, we also used the merged data set to test the prognostic capability of our prognostic genes. As a result, survival analysis shows the hazard ratio (HR) of the two groups stratified by our method is 1.51 (95% confidence interval: 1.30 – 1.76) and the p-value of the log-rank test is 3.73e-08 (Figure 4). Therefore, a conclusion can be drawn that our prognostic genes can not only discriminate the prognostic risk of cancer patients in training data set, but also stratify the patients in independent data sets.

For comparison, the control signature, which contained the most significant genes (40 genes) calculated by the resample method, was also applied to stratify the patients in the six data sets. The results of the control signature in these data sets were shown in Supplementary Table 4. In the training data set and the testing data set which are from TCGA, it is reasonable that the control signature achieved better results because the genes in the control signature are the most significant genes obtained in TCGA. However, in the other four data sets which are independent from TCGA, our prognostic genes outperform the control signature. What is more, the control signature can't distinguish the prognostic risks of cancer patients in two of them (GSE32062 and GSE17260). As we know, the only difference between our prognostic genes and the control signature is that our method concerning the gene dependency relation among genes and DirGenerank algorithm was applied to identify the driver genes in the network. The good performance of our prognostic genes validated that our pipeline can indeed identify the key genes in cancer prognosis.

### Evaluating the independence of prognostic value of the prognostic genes with other clinical variables

In order to evaluate the independence of our selected genes’ prognostic value, we selected all the samples which have the clinical variables of age, grade and stage in TCGA. As a result, 553 samples were obtained. First of all, we performed multivariate Cox regression using risk scores of our prognostic genes, age, stage and grade as co-variables with the death risks in the training data set, testing data set and the entire data set of TCGA. As a result, risk score, age and stage were significant with the death risks of cancer patients in all the three data sets (Supplementary Table 5). In addition, p-values of risk score in the three data sets were 3.76e-21, 0.004 and 2.02e-18 respectively, which outperform the other clinical variables.

**Figure 5:**
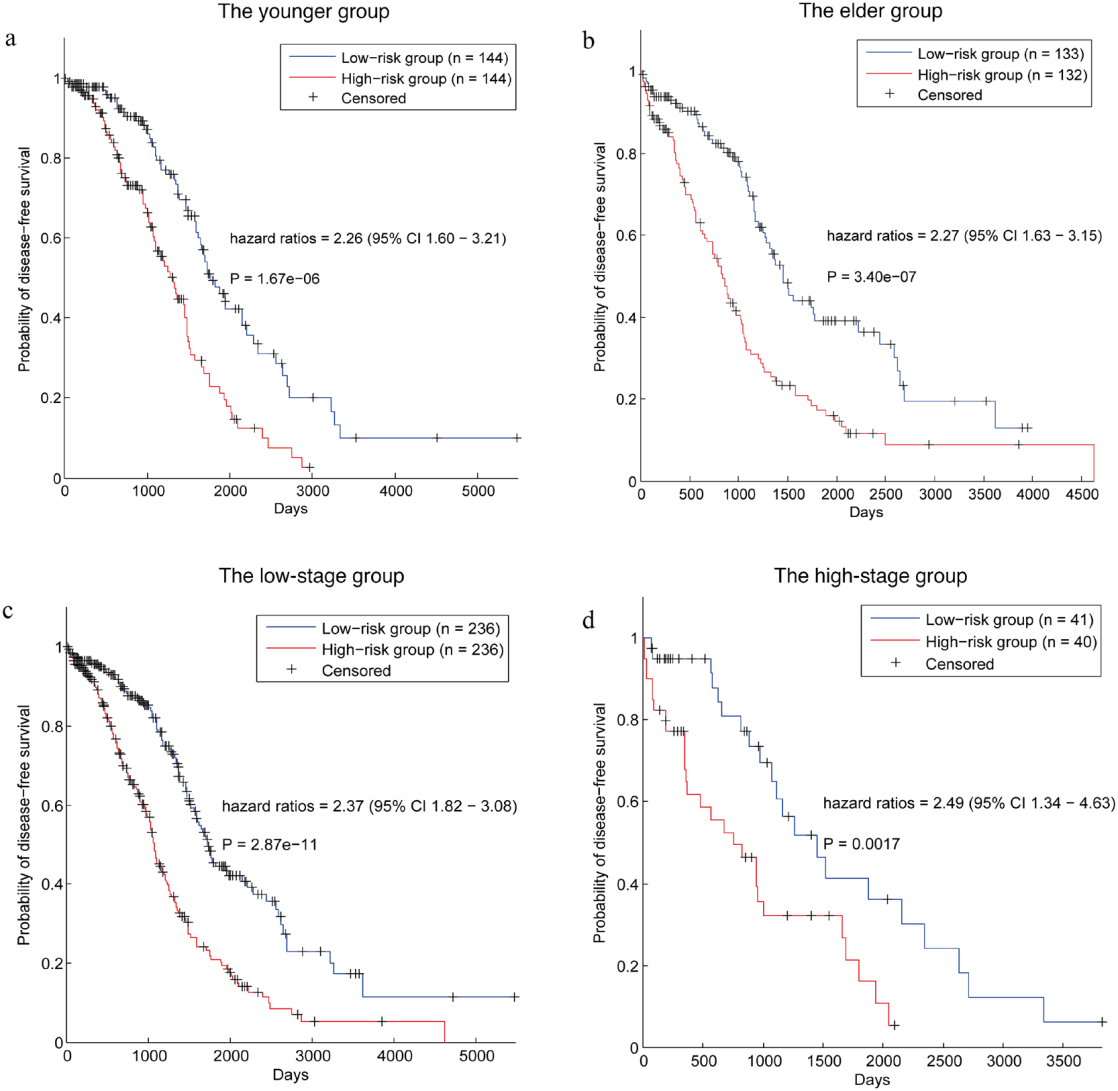
Survival analysis of the patients stratified by age and stage **(a)** Survival analysis of the patients in younger group. **(b)** Survival analysis of the patients in elder group. **(c)** Survival analysis of the patients in low-stage group. **(d)** Survival analysis of the patients in high-stage group.

Because two clinical variables (age and stage) were also significantly correlated with death risks of cancer patients, we performed data stratification analysis based on age and stage in the entire data set of TCGA (We didn't perform this analysis in the training data set and testing data set because the stratified data sets are too small for survival analysis. For example, the high-stage group in testing data set only contained 38 samples). Using the median of the patients’ age (59) as threshold, the data set was divided into a younger group (288 patients) and an elder group (265 patients). The survival analysis was performed in each of the two groups and the results (Figure 5) show our prognostic genes could discriminate the prognostic risks of cancer patients in both the data sets. The hazard ratio of the patients divided by our prognostic genes in the younger group is 2.26 and p-value is 1.67e-06 (Figure 5a). In the meanwhile, hazard ratio is 2.27 and p-value is 3.40e-07 (Figure 5b) in the elder group.

Furthermore, as the number of patients with stage I and stage II is very small, we used stage III as cutoff (stage IV vs. others) to stratify the patients into two data sets, the high-stage group (81 patients) and the low-stage group (472 patients). The prognostic genes were also applied to divide the patients in each data set into two groups according to the risk score. As a result, in the low-stage group, the overall survival time of patients in the low risk group is significantly higher than that of the high risk group, with a hazard ratio of 2.37 and p-value of 2.87e-11 (Figure 5c). In addition, hazard ratio is 2.49 and p-value is 0.0017 (Figure 5d) in the high-stage group.

All these results indicate that the prognostic value of our prognostic genes is independent from other clinical variables.

### Evaluating the stability of the prognostic genes

The prognostic genes identified by our pipeline may be influenced by the selection of different data sets or parameters. For example, the selection of different Human PPI data sets for inferring gene dependency network, ovarian cancer data sets for training and the constant *d* in DirGenerank. Therefore, we used different data sets and parameters to select the prognostic genes and test whether our 40 prognostic genes are stable.

First of all, expect for the PPI data set [33] used in this work, we also used the most famous PPI data set (STRING [34]) to construct the gene dependency network and ranked the prognostic genes based on the new gene dependency network. The ranked genes are shown in Supplementary Table 6. In order to test whether our 40 prognostic genes could be selected as important genes by the new PPI data set, Kolmogorov-smirnov test was applied to test whether our prognostic genes were also on the top of the new ranked genes. As a result, the p-value of the test is 2.80e-24, which indicates our prognostic genes are also important in the new gene list.

In this work, we applied the TCGA to infer gene dependency network and calculated initial importance of the genes. Here, we also used the merged data set, which contains the largest number of samples to run the same pipeline. Supplementary Table 7 shows the gene list ranked by using the new ovarian cancer data set. Kolmogorov-smirnov test was also applied to test whether our prognostic genes were enriched by the top genes in the gene list and p-value of the test is 6.52e-04. This result may illustrate why our prognostic genes are discriminative in the independent data sets.

In the DirGenerank, the constant *d* describes the weight of the gene dependency network. Therefore, different parameter *d* may influence the identification of prognostic genes. Recently, although a few works have proposed methods to determine the optimal parameter in an algorithm [35–38], in this work, for simple, we set *d* as 0.7, which is the same as a previous work did [20]. Furthermore, we also checked whether the prognostic genes are robust with different *d*. As a larger *d* means that the ranked genes are more dependent on the biological network, compared with the initial importance of the genes, we varied the d as 0.50, 0.60, 0.70 and 0.85 (adopted by GOOGLE) in the algorithm and four gene lists were obtained (Supplementary Table 8). The significances of the enrichment of our 40 prognostic genes with the gene lists of other *d* were also evaluated by Kolmogorov-smirnov test. As a result, p-values are all less than 7.20e-35.

From all these results, a conclusion can be drawn that our prognostic genes are stable with the selection of different data sets or parameters in the pipeline.

### Drug screening using the prognostic genes with CMAP

CMAP (The connectivity map) is a famous tool to screen drug [18, 19]. It screens drugs by comparing the up-regulated genes (up signature) and down-regulated genes (down signature) in a disease with the gene expression profiles of tissues stimulated by small molecules. In order to test whether our prognostic genes are good candidates for therapy target, we used the prognostic genes as up signature or down signature to screen drugs using CMAP. Of the 40 prognostic genes, 19 genes with positive cox coefficient were set as up signature and the other 21 genes were set as down signature. As a result, 85 drugs were significant with a p-value < 0.05 (Supplementary Table 9). Among these drugs, 28 drugs (p-value < 0.01) were listed in Table 3.

**Table 3:** Drugs selected by CMAP using prognostic genes

Rank	CMAP name	p-value	Tag
**1**	trichostatin A	0	true
**2**	PHA-00745360	0.00018	unclear
**3**	mephenytoin	0.00022	true
**4**	Gly-His-Lys	0.00026	unclear
**5**	resveratrol	0.0003	true
**6**	quinpirole	0.00034	unclear
**7**	etiocholanolone	0.00052	unclear
**8**	vorinostat	0.00064	true
**9**	aciclovir	0.00127	unclear
**10**	0175029-0000	0.00177	unclear
**11**	GW-8510	0.00223	unclear
**12**	dantrolene	0.00226	false
**13**	irinotecan	0.00246	true
**14**	folic acid	0.00328	true
**15**	midodrine	0.0033	false
**16**	lobelanidine	0.00442	unclear
**17**	alsterpaullone	0.00503	unclear
**18**	tranylcypromine	0.00611	false
**19**	isometheptene	0.00629	false
**20**	Prestwick-857	0.00631	unclear
**21**	morantel	0.00647	unclear
**22**	clebopride	0.00712	unclear
**23**	levomepromazine	0.00722	unclear
**24**	piribedil	0.00859	false
**25**	pentamidine	0.00897	true
**26**	Prestwick-691	0.00937	unclear
**27**	Prestwick-664	0.00985	unclear
**28**	vinblastine	0.00988	true

The first column is the rank of the drugs; the second column is the name of the drugs; the third column describes the p-value of the Kolmogorov–Smirnov test; in the last column, true denotes adaptation disease of the drug contains cancer, and false denotes cancer is not an adaptation disease of the drug.

Among the 28 drugs, 13 drugs are collected in DGIdb (Drug-Gene Interaction database) [39], TTD (Therapeutic target database) [40] or DrugBank [41, 42]. After the investigation of the adaptation diseases of the 13 drugs, 8 drugs could be used as therapy for cancer. Among the drugs in top 10, apart from the ones with unclear adaptation disease, the adaptation diseases of all the other drugs contain cancer. Therefore, it may indicate that our prognostic genes may be used as targets for drugs.

Furthermore, we investigated whether some drugs’ adaptation disease selected by our method was actual ovarian cancer. We screened all the drugs in DGIdb (Drug-Gene Interaction database) [32], TTD (Therapeutic target database) [33] and DrugBank [34, 35] and found 3 drugs (MK-886, MS-275 and Y-27632), of which adaptation disease was ovarian cancer, were contained in CMAP. Among the three drugs, MS-275 and Y-27632 are significant (p-value were 0.034 and 0.048 respectively). Kolmogorov-smirnov test shows the three drugs were significantly enriched on the top of the drug list ranked by CMAP (p-value = 0.0062). In addition, it is reported that trichostatin A, which is the most significant drug (p-value = 0), could be used as treatment for ovarian cancer [43]. All these results could prove the therapy value of our prognostic genes.

## DISCUSSION

Identifying the prognostic genes in cancer is essential not only for the treatment of cancer patients, but also for drug discovery. But it's still a big challenge to select the prognostic genes because the selected genes whose expression levels are statistically related to prognostic risks may be passengers. In this paper, we proposed a new pipeline to identify prognostic genes in cancer (ovarian cancer as a case study in this work). Our pipeline uses gene expression profiles and clinical information (prognostic data) of cancer patients to infer gene dependency network, which could reveal the gene dependency relation in cancer prognosis. After that, resample method was used to evaluate the statistical relation of each gene's expression levels with prognostic risks of cancer patients. Finally, DirGenerank, a modified Pagerank algorithm, was proposed to prioritize genes based on gene dependency network and genes’ statistical relation with prognostic risks of cancer patients.

Analysis of gene dependency network in ovarian cancer shows the network could reveal gene dependency relation in the biological process of cancer. After that, 40 genes were obtained by our pipeline. Functional analysis shows these genes are involved in pathways in cancer, and these genes are also significantly enriched with cancer genes. In addition, the prognostic genes can discriminate the prognostic risks of cancer patients in five data sets, which contain more than 2,000 samples. Furthermore, the prognostic genes are proved to be robust with the selection of different data sets or parameters in the pipeline. And the prognostic values based on these genes are independent from other clinical variables. At last, drug screening using the prognostic genes with CMAP shows the genes in the signature may be drug targets for therapy.

In conclusion, we have proposed a useful pipeline to identify prognostic genes. It needs to be mention that our pipeline could be used not only in the identification of prognostic genes in any disease, but also in the selection of key genes in other biological processes, as long as there are expression profiles of enough samples and phenotype information of these samples.

## MATERIALS AND METHODS

### Data sets and pre-processing

Five ovarian cancer data sets, each of which contains gene expression profiles and clinical information (time to death and status of death) of more than one hundred patients, were collected in this work. Among these data sets, one is from TCGA (The Cancer Genome Atlas) [44], and three are from NCBI (National Center for Biotechnology Information Gene Expression Omnibus) with accession numbers of GSE32062 [45], GSE17260 [46] and GSE26712 [47] respectively. Apart from that, we also used a merged data set containing more than 1000 patients, which was collected from a previous work [32], to validate our method. The gene expression data of the first three data sets were performed with Agilent gene-chips and the other two data sets were performed with Affymetrix gene-chips. The platform of TCGA data set is Agilent G4502A and the platform of the other two Agilent gene-chips is Agilent-014850 Whole Human Genome Microarray 4×44K G4112F. In the meanwhile, the platform of two Affymetrix gene-chips is Human Genome U133A. We mapped the probes to Entrez Gene ID, and the expression levels of the probes for each gene were averaged.

When the data sets were used for survival analysis, the TCGA data set was divided into two parts randomly (used as training data set and testing data set respectively), and the other four data sets were set as independent test sets. The detailed information of these data sets is shown in Table 4. In addition, the prognostic information (days to death, status of death), age, tumor stage and tumor grade of all the patients in TCGA is shown in Supplementary Table 10.

**Table 4:** Ovarian cancer data sets used in this work

Data set	Number of samples	Usage	Site
**TCGA**	300	Training	https://portal.gdc.cancer.gov/
**TCGA**	267	Test	https://portal.gdc.cancer.gov/
**GSE32062**	260	Independent test	https://www.ncbi.nlm.nih.gov/geo/query/acc.cgi?acc=GSE32062
**GSE17260**	110	Independent test	https://www.ncbi.nlm.nih.gov/geo/query/acc.cgi?acc=GSE17260
**GSE26712**	185	Independent test	https://www.ncbi.nlm.nih.gov/geo/query/acc.cgi?acc=GSE26712
**Merged set**	1287	Independent test	http://kmplot.com/analysis/index.php?p=download

It should be mention that the TCGA data set was downloaded from http://tcga-data.nci.nih.gov/tcga/. And the source now is on https://portal.gdc.cancer.gov/.

When we constructed the gene dependency network, the prognostic information and the gene expression in TCGA was discretized. The prognostic information used in this work is the overall survival time and survival status of each patient. If the death of a patient occurred within 1200 days, we set the phenotype as 1; if a patient had an overall survival time of no less than 1200 days, then the phenotype of it was set as 0; otherwise, it was abandoned. For each gene's expression level, if it is higher than the median of the gene's expression levels across all the samples, it was set as 1; otherwise, it was set as 0.

The human PPI (protein-protein interaction data) was downloaded from a previous work [33]. In order to test whether our method is dependent on the selection of PPI, we also downloaded another Human PPI database: STRING [34]. In STRING, the pairs with score of no less than 400 were retained for the next analysis. The cancer genes were obtained from the database Sanger and COSMIC [48]. The drug target information was derived from DGIdb (Drug-Gene Interaction database) [39], TTD (Therapeutic target database) [40] and DrugBank [41, 42].

### Inference of gene dependency network

Based on the hypothesis that the correlation between one gene and the prognostic risks of cancer patients may be dependent on another gene, we proposed a frame to infer gene dependency network [15]. These days, many similarity calculation methods have been widely used in bioinformatcs. For example, in the area of drug-drug similarity [49, 50] or correlation of gene expression data between gene pairs [51]. As the information-theoretic approaches have succeeded in inferring biological networks [51], we used conditional mutual information to infer gene dependency network based on gene expression data and clinical information (days to death and status of death) of ovarian cancer patients (TCGA). The procedure of establishing the gene dependency network is described as follows:

Firstly, the gene expression level of each gene and the clinical information of ovarian cancer patients in TCGA was discretized (Section ‘Data Sets and pre-processing’).

Secondly, for each gene pair in human PPI, we used *CMI* (*conditional mutual information*) to calculate the gene dependency relation of one gene (denoted as *A*) to the other (denoted as *B*). That is, in the context of gene *B*, the mutual information of gene *A*'s expression level and the clinical information *P* (*CMI (A,P|B)*).

Thirdly, to evaluate the significance of the gene dependency relationship of gene *A* to gene *B*, permutation test was used. For each gene pair (*A, B*), we calculated its real *CMI* value using the tool mRMR [52], as described in our previous work [15]. Then the expression levels of gene B across all the patients were randomly permuted, and a new *CMI* value was calculated. After that, the permutation procedure was repeated 1000 times and the 1000 *CMI* values were regarded as the null hypothesis distribution. At last, based on the null hypothesis distribution, p-value of gene dependency relationship of gene *A* to gene *B* was calculated.

Finally, all the significant gene dependency pairs (p-value <= 0.05) were used to construct the gene dependency network. In the network, nodes are genes, and the directed edge (*B*→*A*) represents the mutual information of gene *A* and clinical information is significantly dependent on gene *B*.

### Using resample method to calculate the prognostic capability of each gene

We used a resampling method to evaluate the prognostic capability of each gene, which would be used as an input of the DirGenerank algorithm. The resampling method is shown as follow:

Firstly, the gene expression data and prognostic information of 300 patients was randomly selected from TCGA, which is used as training data set.

Secondly, 90% of all the 300 samples were randomly chosen. For each gene, we used Cox proportional hazards regression to calculate the relation between the gene expression levels and the death risk (death time and death status) across the selected patients.

Thirdly, step 2 was repeated 400 times, and the times of each gene whose Cox p-value was less than 0.05 can describe the stability of the gene's prognostic capability. The stability of each gene was used to characterize the prognostic capability of it. In addition, the Cox coefficients of each gene in the 400 runs were averaged as the final Cox coefficient of the gene.

### Using DirGenerank to select essential genes

Pagerank [16] was invented to rank the important web pages on the internet through the links among these web pages. In a previous work, the Generank algorithm [17], a modified Pagerank algorithm, succeeded in selecting genes from biological network. The Generank was proposed for undirected network. We extended it to direct network, which is called DirGenerank. The algorithm is described as the following equation:

$$r_{j}^{n}=(1-d){\text{imp}}_{j}+d\sum_{i=1}^{N}\frac{w_{ij}r_{i}^{n-1}}{{\text{deg}}_{i}}.$$

Where, $r_{j}^{n}$ is the importance of gene *j* after *n* iterations and $r_{i}^{n-1}$ the importance of gene *i* after *n-1* iterations; $imp_{j}$ is the initial importance of gene *j*, and in our work, we set $imp_{j}$ as the prognostic capability calculated by the resample method; $w_{ij}$ describes the gene dependency relation between gene *i* and gene *j*, that is, if gene *i* is significantly dependent on gene *j*, then $w_{ij}$ = 1, otherwise, $w_{ij}$ = 0; ${\text{deg}}_{i}$ is the out-degree of gene *i*, in another word, how many genes are significantly dependent on gene *i*; *N* is the number of genes in gene dependency network; *d* (*0 ≤ d < 1*) is a constant, which describes the weight of the gene dependency network. If *d* is zero, it means the importance of the gene equals the initial importance, while *d* is close to 1, it means the importance of the gene almost completely dependent on the gene dependency network. In this work, we set it as 0.70. The iteration of the algorithm stops until ε < 0.00001, while $\varepsilon =\;|r_{j}^{n}-r_{j}^{n-1}|$ and |.| is one-norm. The code of the algorithm is submitted in supplementary file.

### Calculation of the prognostic risk

After obtaining the prognostic signature, we applied a method similar with GGI [53] to calculate the prognostic risk of each patient:

$$\text{Risk Score}=\sum x_{i}-\sum x_{j}.$$

Where $x_{i}$ is the expression level of gene whose Cox coefficient is positive, while $x_{j}$ is the expression level of gene whose Cox coefficient is negative. After that, the patients in each data set were divided into two groups: the patients with the risk score not higher than a threshold, which is equal to the median of the risk scores of all the patients in the data set, were set as low risk group; otherwise, the patients were set as high risk group. At last, the log rank test was performed to test whether there is significant difference of the death risk between the two groups divided by our method.

### Enrichment analysis

GSEA [54] was used to investigate the functions (KEGG pathway) of the prognostic signature. We applied hypergeometric test to test whether the intersection of cancer genes with the prognostic genes is significant, which was calculated as follow:

$$p-\text{value}=F(x/M,K,N)=1-\sum_{i=0}^{x-1}\frac{\left(\begin{array}{l} K\\ i \end{array}\right)\left(\begin{array}{l} M-K\\ N-i \end{array}\right)}{\left(\begin{array}{l} M\\ N \end{array}\right)}.$$

Where *x* is the size of the intersection set, *K* is the number of our prognostic genes, *N* is the number of the cancer genes and *M* is the size of universal set.

### Network topology and visualization

We used Cytoscape 3.2.0 to visualize the gene dependency network and the Network Analyzer plug-in for Cytoscape [55] was applied to analyze the topology of the network.

## SUPPLEMENTARY MATERIALS FIGURES AND TABLES
















