# Supplementary material for: Identifying prognostic signature in ovarian cancer using DirGenerank
Source: Oncotarget. 2017 May 25;8(28):46398–413. doi: 10.18632/oncotarget.18189 (PMC5542276; doi:10.18632/oncotarget.18189)
Supplement: Supplementary file 1 [file oncotarget-08-46398-s001.pdf]

Identifying prognostic signature in ovarian cancer using DirGenerank

SUPPLEMENTARY MATERIALS

Matlab code of DirGeneRank

```
%%%%%%%%%%%%%%%%%%%%%%%
function r = DirGeneRank(W,ex,d)
%function r = DirGeneRank(W,ex,d)
%
% DirGeneRank is a modification of the PageRank algorithm using in direct network.
% input data is W: connectivity matrix (zero/one)
% ex: vector of value(non-negative), which describes the
% importance of all the genes
% d: parameter in algorithm
%
% output is r: vector of rankings
%
% March 09/2017
%
ex = abs(ex);
norm_ex = ex/max(ex);
b = (1-d)*norm_ex';
degrees = (sum(W,2))';
ind = degrees == 0;
degrees(ind) = 1;
D1 = (1./degrees);
c = b+d*norm_ex'.*D1*W;
while(1)
c2 = b+ d*c.*D1*W;
if sum(abs(c2-c))<0.00001
break;
end;
c=c2;
end;
```

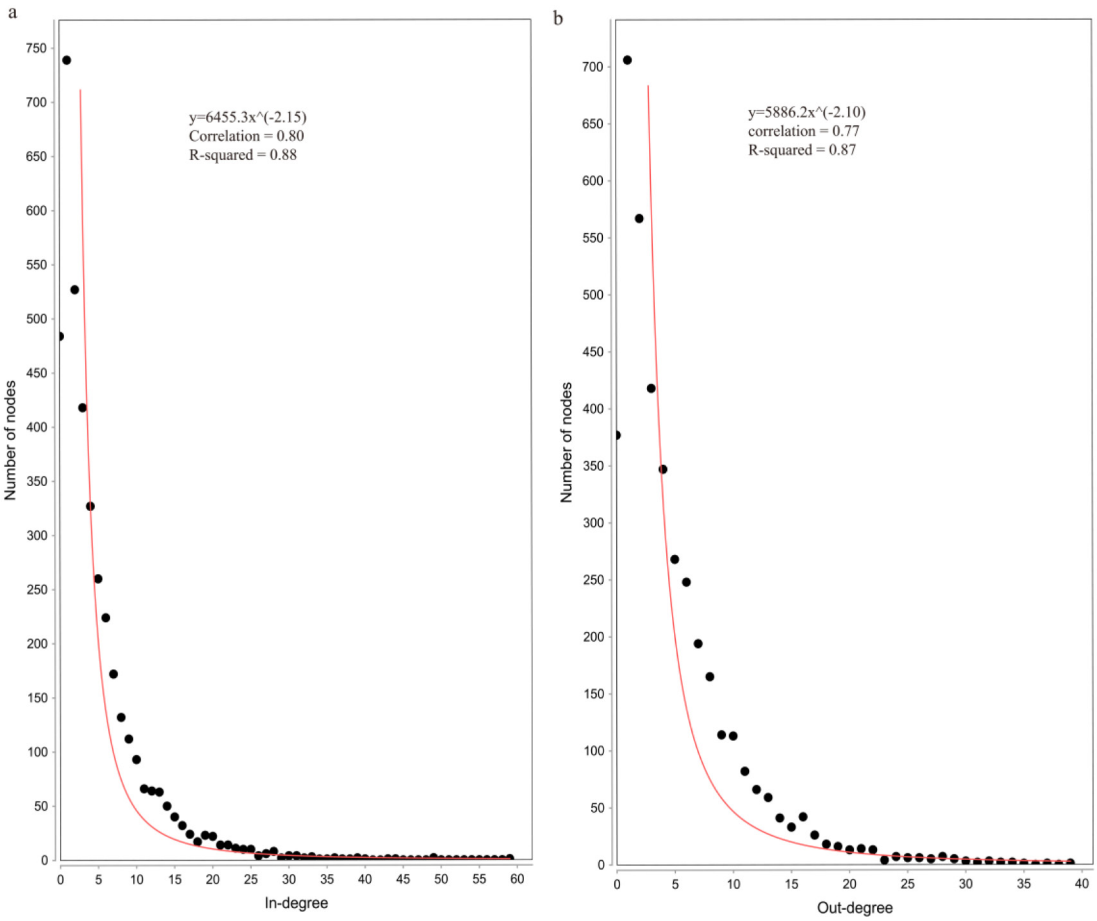

Supplementary Figure 1: The power law-fit of the in-degree (a) and out-degree (b).

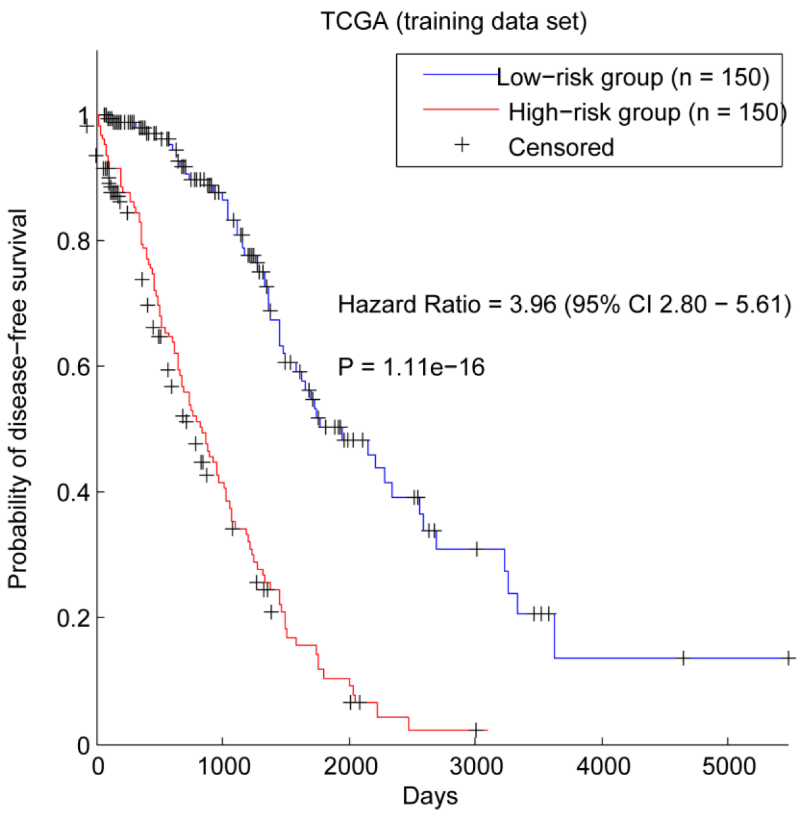

Supplementary Figure 2: Survival analysis of our prognostic genes in train data set.

Supplementary Table 1: The gene dependency network

See Supplementary File 1

Supplementary Table 2: Result of the resample method

See Supplementary File 2

Supplementary Table 3: The control signature

Gene ID	Gene symbol	Cox coefficient	Stability
125	ADH1B	0.23918	400
344	APOC2	0.295606	399
1041	CDSN	0.391074	399
1373	CPS1	0.33927	400
1381	CRABP1	-0.13177	399
2091	FBL	0.374389	400
2250	FGF5	-0.54169	400
3082	HGF	0.36147	400
3207	HOXA11	-0.3317	400
3291	HSD11B2	-0.3044	400
3420	IDH3B	0.461632	400
4025	LPO	-0.35354	400
5320	PLA2G2A	0.215706	400
5530	PPP3CA	0.419775	400
6217	RPS16	0.34258	400
7082	TJP1	0.45589	400
7092	TLL1	0.455569	400
7538	ZFP36	0.187581	400
23051	ZHX3	0.414173	400
24147	FJX1	-0.42383	400
25824	PRDX5	-0.50188	400
27329	ANGPTL3	-0.46493	400
27335	EIF3K	0.360984	400
56259	CTNNBL1	0.523634	400
56270	WDR45L	-0.36113	400
79668	PARP8	-0.39747	400
79799	UGT2A3	-0.38019	400
80028	FBXL18	0.503824	400
80146	UXS1	-0.3467	400
80195	C10orf57	-0.51189	400
90134	KCNH7	1.292207	400
90522	YIF1B	0.47367	400
91419	XRCC6BP1	-0.52322	400
140460	ASB7	-0.33388	400
146057	TTBK2	0.79329	400
160728	SLC5A8	-0.46904	400
163131	ZNF780B	0.261745	400
163732	CITED4	-0.27245	400
283579	C14orf178	-0.43847	400
440279	UNC13C	-0.3239	400

Supplementary Table 4: Survival analysis of the patients divided by the control signature

	Hazard ratio	95% CI -	95% CI +	Log-rank p-value
Training data set	4.43	3.09	6.35	0
Testing data set	1.59	1.13	2.22	0.0034
GSE17260	1.17	0.75	1.84	0.28
GSE32062	0.98	0.68	1.40	0.47
GSE26712	1.66	1.17	2.35	0.0027
Merging data set	1.49	1.28	1.73	8.76E-08

Supplementary Table 5: P-value of multivariate Cox regression of risk score, age, grade and stage with the prognostic risk

	Risk score	Age	Grade	Stage
**Training data set**	3.76e-21	0.0046	0.94	0.026
**Testing data set**	0.004	0.058	0.20	0.027
**Entire data set**	2.02e-18	2.75e-04	0.18	0.0038

Supplementary Table 6: The ranked gene list calculated using STRING

See Supplementary File 3

Supplementary Table 7: The ranked gene list calculated using the merged data set

See Supplementary File 4

Supplementary Table 8: The ranked gene lists with different d

See Supplementary File 5

Supplementary Table 9: Drugs screened by CMAP

See Supplementary File 6

Supplementary Table 10: The clinical information of cancer patients in TCGA

See Supplementary File 7
